# Elevated Pretreatment Plasma Oncostatin M Is Associated With Poor Biochemical Response to Infliximab

**DOI:** 10.1093/crocol/otz026

**Published:** 2019-08-19

**Authors:** Phillip Minar, Christina Lehn, Yi-Ting Tsai, Kimberly Jackson, Michael J Rosen, Lee A Denson

**Affiliations:** Division of Gastroenterology, Hepatology, and Nutrition, Department of Pediatrics, University of Cincinnati College of Medicine, Cincinnati Children’s Hospital Medical Center, Cincinnati, OH

**Keywords:** biomarkers, Crohn disease, companion diagnostic, neutrophil CD64, pediatric

## Abstract

**Background:**

We hypothesized that elevations of plasma Oncostatin M (OSM) would be associated with infliximab nonresponse.

**Methods:**

Plasma OSM was measured in Crohn disease patients pre-infliximab with biochemical response (>50% reduction in fecal calprotectin) as the primary outcome.

**Results:**

The median OSM in biochemical responders was 86 (69–148) pg/mL compared with 166 (74–1766) pg/mL in nonresponders (*P* = 0.03). Plasma OSM > 143.5 pg/mL was 71% sensitive and 78% specific for biochemical nonresponse (area under the curve 0.71). Early biochemical nonremission was also associated with an elevated neutrophil CD64 expression (odds ratio 8.9, *P* = 0.011).

**Conclusions:**

Elevated preinfliximab plasma OSM and nCD64 surface expression were both associated with poor biochemical outcomes.

## Lay Summary

Pretreatment elevations in plasma Oncostatin M and whole blood neutrophil CD64 are associated with unfavorable early and late treatment outcomes to infliximab in patients with Crohn disease.

## INTRODUCTION

Therapeutic options for moderate-to-severe Crohn disease (CD) include monoclonal antibodies (mAb) that antagonize circulating or tissue-bound inflammatory cytokines and leukocyte recruitment pathways. Following the pivotal REACH study,^1^ mAb’s targeting tumor necrosis factor-alpha (anti-TNF) have become and remain the most commonly used first-line biologic agents for children with moderate-to-severe CD. Despite high rates of clinical response to anti-TNF (75%–88.4%),^1, 2^ many children and adults continue to experience symptom flares and serious disease complications as rates of mucosal healing range between 36% and 46% with only 19%–36% achieving deep remission (both clinical remission and mucosal healing).^3, 4^ Although rates of deep remission are likely to improve with increased utilization of personalized anti-TNF dosing regimens^5^ and effective dose optimization strategies following proactive therapeutic drug monitoring,^6^ a sizable percentage of children will continue to fail anti-TNF despite adequate drug exposure.

Despite the heterogeneity of CD phenotypes and a potentially aggressive course of inadequately treated CD, treatment selection is currently based on clinical factors that poorly define CD subtype (mainly, the potential for a stricturing or fistulizing phenotype). Previous studies to evaluate baseline predictors of anti-TNF nonresponse have found that older age,^3^ very early onset disease,^4^ history of smoking,^7^ and history of a CD-related surgery^3^ are clinical risk factors for poor response. Further studies have found that c-reactive protein,^8^ hypoalbuminemia, and low TREM1^9^ are associated with poor response. As new biologics are available, there has been renewed interest for an anti-TNF companion diagnostic as associations between molecular inflammatory signatures (tissue-based) and anti-TNF response have also been discovered.^10–12^

In a recent publication, West et al found that Oncostatin M (*OSM*) and OSM receptor (*OSMR*) were highly expressed in the inflamed intestinal tissue in newly diagnosed, treatment-naive pediatric CD patients.^12^ Moreover, hierarchical clustering was performed to discover additional cytokines and chemokines that were associated with a high expression of *OSM* (referred to as the OSM^high^ module). Remarkably, West et al found that patients within the OSM^high^ module were largely (90%) infliximab refractory with a significant correlation between elevated *OSM* and need for surgery.^12^ The authors, however, did not report on plasma OSM and anti-TNF outcomes.

Our group has previously found that the ileal and rectal mRNA expression of Fcγ receptor IA (*FCGRIA*) was up to 3-fold higher in new diagnosis, treatment-naive CD compared with controls.^13^ We have subsequently found similar elevations in peripheral blood neutrophil FCGRIA (nCD64) in treatment-naive pediatric CD patients.^13, 14^ We also found that nCD64 expression correlates with endoscopic severity,^14^ while elevations in nCD64 is a significant risk factor for treatment relapse in silent (asymptomatic) CD.^15^ Although intestinal *FCGRIA* mRNA expression was found to be upregulated in infliximab nonresponders,^16^ peripheral blood nCD64 expression and anti-TNF outcomes have not been previously investigated.

With a goal to develop a noninvasive, blood-based companion diagnostic for anti-TNF refractory CD, we hypothesized that increases in plasma OSM would also be associated with early and late anti-TNF nonresponse. Moreover, we explored the association between nCD64 and early/late anti-TNF outcomes.

## MATERIALS AND METHODS

### Patient Recruitment

To test our hypothesis, we performed a pilot investigation of CD patients enrolled in the Clinical and Molecular Signature to Predict Response to Anti-TNF Therapy in Pediatric IBD (PROSE) study. PROSE is a single-center, inception cohort of children and young adults (≤22 y old) with inflammatory bowel disease (IBD) who enrolled immediately prior to starting infliximab at Cincinnati Children’s Hospital Medical Center between July 2014 and October 2018. Participants were prospectively monitored for treatment response with longitudinal biospecimens collected for 1 year. All patients enrolled were anti-TNF naive, and their infliximab dose and frequency were determined by the treating provider.

### Study Outcomes

The primary outcome measure was pretreatment plasma OSM concentration and the start of maintenance (week12 [W12]) biochemical response defined as >50% reduction from the patient’s baseline fecal calprotectin.^17^ Secondary outcomes included pretreatment plasma OSM concentration or nCD64 and biochemical remission at either W12 or week52 (W52; fecal calprotectin < 250 µg/g^18, 19^). Clinical remission was assessed with the weighted pediatric CD activity index (wPCDAI) and defined as a wPCDAI < 12.5.^20^ The mathematically wPCDAI combines subjective clinical evaluation (abdominal pain, stool frequency, and general well-being) and laboratory tests (albumin and erythrocyte sedimentation rate) with physical exam assessments (weight, perirectal disease, and evaluation of extraintestinal manifestations) and has been shown to correlate with mucosal inflammation.^21^ For analysis of the primary outcome, we excluded patients who (a) failed to provide stools samples prior to either infusion1/infusion4 or (b) had a baseline fecal calprotectin < 250 µg/g.

### Biologic Assays

Plasma OSM concentrations were determined by an enzyme-linked immunosorbent assay (ELISA; Thermo Scientific, MA) from blood samples collected at infusion1 and infusion4. The ELISA has an upper detection limit of 1000 pg/mL, lower detection limit of 1 pg/mL at 1:2 dilution, and an intra-assay coefficient variation (CV) < 12%. Whole blood nCD64 was measured by quantitative flow cytometry on a FACSCalibur (BD Biosciences, San Jose, CA) using the Leuko64 assay kit (Trillium Diagnostics, Brewer, ME). The kit includes fluorescent beads and antibodies to CD64 and CD163. The lymphocyte, monocyte, and granulocyte populations are defined by their forward and side scatter characteristics with CD163 staining to further define the monocyte population. The neutrophil CD64 index is the result of the ratio of the mean fluorescent intensity of the granulocytes to that of the calibration beads.

Fecal calprotectin was measured from stool samples collected prior to infusion1 and infusion4 utilizing an ELISA kit with an intra-assay CV of 2.6%–10.5% (Buhlmann, Switzerland).^22^ Trough infliximab concentrations were determined with IDKmonitor (Immundiagnostik, Germany) from stored plasma samples collected immediately prior to the fourth infliximab infusion. We did not test for the presence of antibodies to infliximab. The infliximab ELISA has an upper detection limit of 45 µg/mL, lower detection limit of 0.7 µg/mL at 1:200 dilution, and an intra-assay CV of 1.8%–9.7%.^23^

### Statistical Analysis

Continuous variables are represented as means with SD or as medians with interquartile range (IQR) depending on data distribution. Plasma OSM concentrations at infusion1 were compared between biochemical responders and nonresponders using the Mann–Whitney test. The optimal pre-infliximab OSM concentration cut point was determined for biochemical nonresponse using the Youden index from the receiver-operating characteristic (ROC) curve. The area under the ROC curve (AUC) with 95% confidence intervals (CI), sensitivity, specificity, positive predictive value, and negative predictive value for OSM concentrations were determined for biochemical nonresponse. We utilized this new cut point to define OSM^low^ and OSM^high^. Rates of biochemical response and remission at W12 and W52 were compared by OSM status (low/high) using the Fisher exact test. Pre-infliximab (baseline) categorical variables were assessed for significance for biochemical response and remission using a univariate logistic regression analysis. To reduce the risk of overfitting, the multivariate regression analysis only evaluated OSM and nCD64. Finally, a Kaplan–Meier survival analysis was performed to evaluate the association between the intensity of the blood biomarkers (OSM^high^/nCD64^high^) and time to an unfavorable treatment outcome (surgery or discontinuation of infliximab). A *P* value of <0.05 was considered statistically significant. All statistical analyses was performed using PRISM version 7 (GraphPad, San Diego, CA) and R version 3.4.3 (R Development Core Team, Austria).

### Ethical Considerations

The PROSE study was approved by the Institutional Review Board at Cincinnati Children’s Hospital Medical Center.

## RESULTS

Plasma OSM was measured immediately prior to the first infliximab infusion from 40 consecutively enrolled anti-TNF naive CD patients. The mean (SD) age was 13 (4) years old, with 35% female and 5% had a preceding CD-related surgery prior to starting infliximab. Sixty-five percent of the cohort started infliximab within 90 days of diagnosis and the median (IQR) pre-infliximab fecal calprotectin was 1519 µg/g (767–2501). One patient was receiving an immunomodulator in combination with infliximab during induction. Additional patient demographics and disease characteristics are listed in Table 1.

**TABLE 1.** Clinical Characteristics and Baseline Laboratory Results

Number of patients, N	40
Female, n (%)	14 (35)
White race, n (%)	36 (90)
Age at first infusion, y (mean, SD)	13 (4)
Disease duration, d (median, IQR)	43 (18–240)
<90 d, n (%)	26 (65)
Previous surgery, n (%)	2 (5)
Concomitant IMM, n (%)	1 (2.5)
Concomitant prednisone, n (%)	20 (50)
Crohn location	
Ileal only, n	4
Colon only, n	4
Ileocolonic, n	32
Crohn behavior	
Inflammatory, n	32
Stricturing, n	6
Penetrating, n	1
Both stricturing/penetrating, n	1
Perianal Crohn, n (%)	10 (25)
BMI, kg/m^2^ (median, IQR)	17.3 (15.2–20.9)
BMI *z*-score (mean, SD)	−0.71 (1.2)
wPCDAI (mean, SD)	43 (28)
ESR, mm/h (median, IQR)	18 (10–26)
CRP, mg/dL (median, IQR)	1.1 (0.42–2.2)
Albumin, g/dL (mean, SD)	3.4 (0.5)
Neutrophil CD64 index (median, IQR)	1.3 (0.92–1.84)
Fecal calprotectin, µg/g (median, IQR)	1519 (767–2501)

IMM, immunomodulator; BMI, body mass index; ESR, erythrocyte sedimentation rate; CRP, c-reactive protein.

### Elevated Plasma OSM Is Associated With Early Biochemical Nonresponse

**FIGURE 1.**
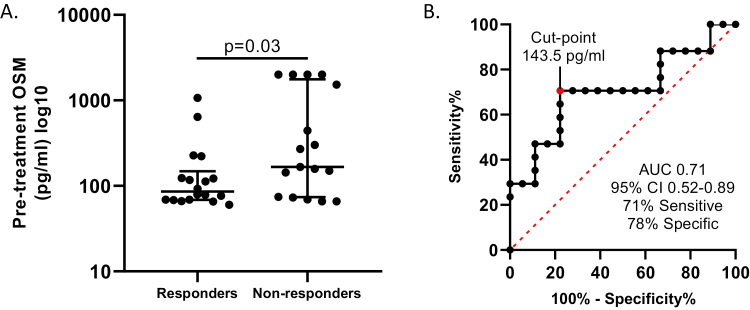
(A) Pre-infliximab plasma OSM concentrations (pg/mL) was evaluated between biochemical responders (>50% reduction in baseline fecal calprotectin) and nonresponders with the Mann–Whitney test (data are log-transformed). (B) Receiver-operating characteristic analysis was then performed to identify the pre-infliximab OSM cut point (Youden index) that was associated with biochemical nonresponse.

The primary analysis was performed from the 35 patients who provided fecal samples prior to starting infliximab and prior to the first maintenance dose. We found 51.4% (18/35) and 34.3% (12/35) of the cohort achieved biochemical response and biochemical remission at W12, respectively. The median pre-infliximab plasma OSM was 86 pg/mL (69–148) in biochemical responders and 166 pg/mL (74–1766) in biochemical nonresponders (*P* = 0.03, Fig. 1A). By performing a ROC curve analysis and subsequently calculating the Youden index, we established a pre-infliximab OSM > 143.5 pg/mL was 71% sensitive and 78% specific with a 75% positive predictive value and a 74% negative predictive value for primary biochemical nonresponse (AUC 0.71, 95% CI 0.52–0.89, Fig. 1B).

The median pre-infliximab plasma OSM was 78 pg/mL (68–199) in biochemical remitters (fecal calprotectin < 250 µg/g) compared with 150 pg/mL (74–444) in nonremitters (*P* = 0.066). In contrast to biochemical responders, the pre-infliximab OSM cut point that distinguished early (W12) biochemical remitters from nonremitters was 117 pg/mL (AUC 0.69, 95% CI 0.5–0.89).

### OSM^low^ Is Associated Higher Rates of Biochemical and Clinical Remission

**FIGURE 2.**
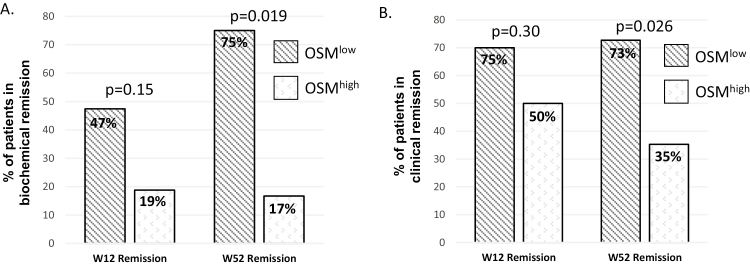
Study participants were either classified as OSM^high^ or OSM^low^ based on their pretreatment plasma OSM level (≥143.5 pg/mL = OSM^high^). (A) OSM^low^ patients had a higher rate of W52 biochemical remission (fecal calprotectin <250 µg/g) and (B) clinical remission (wPCDAI < 12.5) compared with OSM^high^. Rates of remission were compared by the Fisher exact test.

Utilizing the above cut point for early biochemical response, we defined OSM^low^ as a pre-infliximab OSM < 143.5 pg/mL. We found no difference in rates of early (W12) biochemical and clinical remission between OSM^low^ and OSM^high^ patients. At 1 year, however, OSM^low^ patients had significantly higher rates of both W52 biochemical and clinical remission (Fig. 2). In a subset of these 35 patients who also had a fecal calprotectin collected at W52 (n = 20), we found the median pre-infliximab plasma OSM was 77 pg/mL (66–117) in W52 biochemical remitters compared to a median OSM of 444 pg/mL (186–2001) in nonremitters (*P* = 0.004). The cut point for baseline OSM that was associated with W52 biochemical nonremission was >222 pg/mL (AUC 0.88, 95% CI 0.72–1.0, 77% sensitive, 100% specific).

### Elevated Pretreatment nCD64 Is Associated With Biochemical Nonremission

**FIGURE 3.**
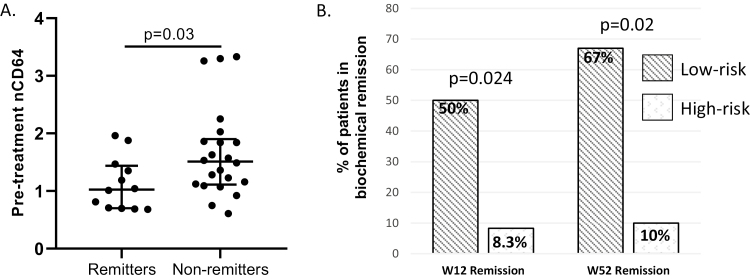
(A) We evaluated pre-infliximab whole blood neutrophil CD64 surface expression (nCD64) between biochemical remitters (fecal calprotectin < 250 µg/g) and nonremitters at the end of induction with the Mann–Whitney test. (B) Study participants were classified as high risk (OSM^high^/nCD64^high^) based on their pretreatment plasma OSM and nCD64 concentrations. High-risk patients had lower rates of biochemical remission at the end of induction (W12) and at W52 compared with the low-risk group. Rates of remission were compared by the Fisher exact test.

We found the median (IQR) nCD64 was 1.03 (0.7–1.4) in early biochemical remitters compared with 1.5 (1.1–1.9) in biochemical nonremitters (*P* = 0.03, Fig. 3A). ROC curve analysis and subsequent Youden index defined nCD64^high^ as 1.07 (AUC 0.73, 95% CI 0.54–0.92). Although there was no correlation between OSM and nCD64 (Spearman *r* = 0.23, *P* = 0.17), patients with a combination of OSM^high^ and nCD64^high^ (classified as “high risk”) was associated with poor early and late biochemical outcomes (Fig. 3B). The high-risk classification included 33% of the cohort.

### Baseline Predictors of Early Biochemical Outcomes

Baseline disease characteristics between OSM^low^ and OSM^high^ patients were similar, with only the pre-infliximab BMI *z*-score statistically different (Table 2). Next, we evaluated pretreatment predictors of poor (early) biochemical outcomes using a univariate regression analysis. After evaluating multiple pretreatment predictors of early nonresponse (Table 3), we found baseline hypoalbuminemia (≤3.5 g/dL; odds ratio [OR] 6.2) and OSM^high^ (>143.5 pg/mL; OR 8.4) were significantly associated with biochemical nonresponse. Pre-infliximab hypoalbuminemia (OR 19, *P* = 0.002), nCD64^high^ (OR 8.9, *P* = 0.011), and OSM^high^/nCD64^high^ (OR 11, *P* = 0.034) were associated with biochemical nonremission. In a multivariate regression evaluating the relationship between biochemical nonremission and nCD64 and OSM, only baseline nCD64^high^ (*P* = 0.017) was independently associated with a pre-maintenance (W12) fecal calprotectin > 250 µg/g.

**TABLE 2.** Comparison of Baseline Disease Characteristics and Laboratory Tests Between Pre-infliximab OSM^low^ (<143.5 pg/mL) and OSM^high^

Baseline Characteristics	OSM^low^ (n = 23)	OSM^high^ (n = 17)	*P*
Age at start of infliximab, y (mean, SD)	13.6 (3.7)	12.8 (4.7)	0.57
Time with Crohn, d (median, IQR)	65 (25–226)	35 (14–280)	0.50
BMI *z*-score (mean, SD)	−1.0 (1.2)	−0.24 (1.1)	0.041
Surgery before anti-TNF, n (%)	2 (8.7%)	0	0.50
Starting infliximab dose, mg/kg (median, IQR)	5.9 (5–6.8)	6.3 (5.8–9.2)	0.08
wPCDAI (median, IQR)	40 (20–60)	40 (18–74)	0.59
CRP, mg/dL (median, IQR)	0.71 (0.28–1.6)	1.4 (0.95–3.4)	0.062
Albumin, g/dL (mean, SD)	3.4 (0.4)	3.3 (0.5)	0.21
Fecal calprotectin, µg/g (median, IQR)	1383 (743–2501)	1656 (817–2501)	0.38

BMI, body mass index; CRP, c-reactive protein.

**TABLE 3.** Pretreatment Predictors of Early (Premaintenance) Biochemical Outcomes

Baseline Characteristic	Odds Ratio	95% CI	*P*
Biochemical nonresponse (<50% reduction from baseline fecal calprotectin)			
≤10-y-old starting infliximab	1.9	0.44–9.1	0.40
wPCDAI > 40	0.9	0.23–3.4	0.88
ESR ≥ 20 mm/h	1.5	0.33–7.5	0.58
CRP ≥ 5 mg/dL	5.5	0.72–115	0.15
Albumin ≤ 3.5 g/dL	6.2	1.2–48	0.042
OSM^high^ (≥143.5 pg/mL)	8.4	2–43	0.006
nCD64^high^ (>1.07)	2.8	0.61–15	0.21
OSM^high^ + nCD64^high^	13.3	2.6–106	0.005
Biochemical nonremission (fecal calprotectin > 250 µg/g)			
Albumin ≤ 3.5 g/dL	19	3.4–165	0.002
OSM^high^ (≥143.5 pg/mL)	3.9	0.90–21	0.084
nCD64^high^ (>1.07)	8.9	1.8–55	0.011
OSM^high^ + nCD64^high^	11	1.7–219	0.034

CRP, c-reactive protein; ESR, erythrocyte sedimentation rate.

### Delta OSM and nCD64 During Induction

**FIGURE 4.**
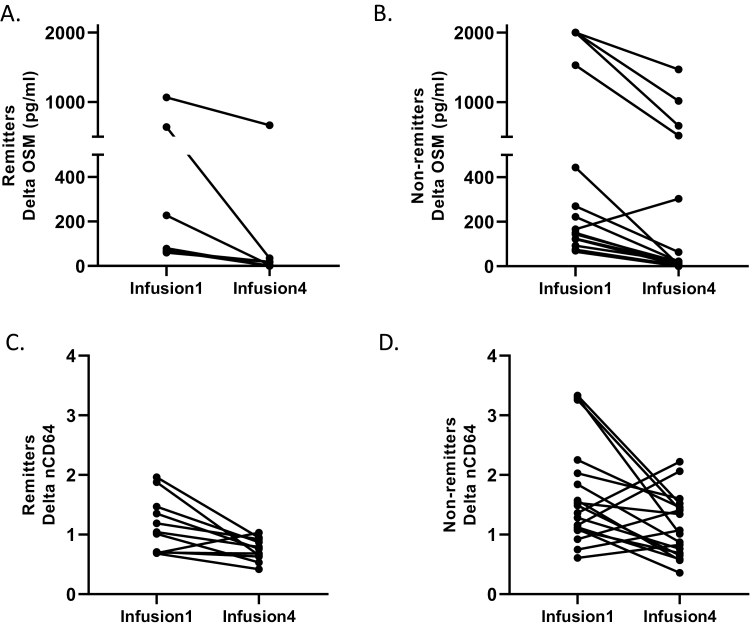
Repeat plasma OSM was available in 30/35 patients that were assessed for the primary outcome with a repeat nCD64 available in 29/35 patients. (A) The absolute change (delta) in plasma OSM between pre-infliximab (infusion1) and pre-maintenance (infusion4) in patients with early biochemical remission and (B) biochemical nonremission. The one patient with an increase in plasma OSM from baseline to W12 had surgery for an ileal stricture 4 months after starting infliximab. (C) The absolute change in nCD64 between pretreatment and pre-maintenance in patients with early biochemical remission and (D) biochemical nonremission.

We found the median reduction (percent change) in OSM from pretreatment to pre-maintenance was higher in biochemical remitters compared with nonremitters (*P* = 0.09). Figure 3A and B demonstrates the change in plasma OSM from baseline (infusion1) to pre-maintenance (W12) in remitters and non-remitters respectively. Plasma OSM was undetectable by the end of induction in 45.5% of the biochemical remitters compared to 21.1% of nonremitter (*P* = 0.23). It is noteworthy that all 6 (100%) patients with an W12 OSM > 143.5 pg/mL (OSM^high^) had evidence of active CD at W52. The one patient with an increase in plasma OSM from baseline to W12 went on to have an ileocecal resection for an ileal stricture 4 months after starting infliximab. We also found that the median reduction (infusion1 to infusion 4) for nCD64 was higher in biochemical responders compared with nonresponders (*P* < 0.05). Although there was no difference in the delta nCD64 percent reduction between remitters and nonremitters (*P* = 0.84), Figure 4C and D demonstrates an overall lower baseline nCD64 in remitters compared with nonremitters.

### Additional Outcomes

One year from starting infliximab, 87.5% of the cohort was receiving infliximab while 12.5% had a CD-related surgery. In a survival curve analysis, we found there was no association between OSM, nCD64 or the combination OSM/nCD64 status and time to a poor outcome (time to surgery and/or time to infliximab discontinuation; data not shown).

As expected, we found biochemical responders and remitters had higher median infliximab concentrations prior to the fourth infusion compared with nonresponders (*P* = 0.048) and nonremitters (*P* = 0.024, Supplementary Fig. 1A and B), respectively. We did not find a difference in the median (IQR) W12 infliximab concentrations between pretreatment OSM^low^ and OSM^high^ patients (Supplementary Fig. 1C).

## DISCUSSION

Approximately 80% of pediatric CD patients treated with anti-TNF will have an early clinical response.^1, 2^ However, recent data strongly support the attainment of mucosal healing as a major treatment target in pediatric and adult-onset CD. In pediatric CD, rates of mucosal healing after 1 year of anti-TNF range from 39% to 63%.^24, 25^ Rates of mucosal healing in adults with CD receiving optimized anti-TNF dosing regimens range from 27% to 40%^5, 26^ further indicating a larger than expected subset of anti-TNF responders in both pediatric and adult-onset CD. With novel CD therapies now available, there is a critical need for the development of a companion diagnostic for all biologics (including anti-TNF) to provide guidance to clinicians for the initial treatment selection as long-term efficacy may be affected by the first biologic exposure.^27^ Respectively, West et al and Wojtal et al found increased intestinal expression of *OSM*^12^ and *FCGRIA*^16^ were associated with anti-TNF nonresponse. In our study, we found that elevations in pretreatment plasma OSM and whole blood nCD64 were associated with unfavorable (early and late) biochemical outcomes to infliximab.

OSM is part of the IL-6 cytokine family that following binding to the OSM receptor (OSMR) functions to induce downstream signaling pathways such as signal transducer and activator of transcription 3, mitogen-activated protein kinase, and phosphatidylinositol-3-kinase.^12^ Functional processes of OSM include a role in the inflammatory response, hematopoiesis, tissue remodeling (liver repair, cardiac tissue remodeling), and osteoclastogenesis.^28^ Overproduction of OSM has been associated with skin and lung inflammation, atherosclerosis and several forms of cancer.^28^ West et al identified expression of *OSM* and *OSMR* from intestinal mucosal biopsies is increased in active CD and ulcerative colitis (UC).^12^ Utilizing data derived from Gene Expression Omnibus (GEO) data sets (162 CD and 42 controls),^29^ West et al reported intestinal *OSM* was especially enriched in CD patients with deep mucosal ulcerations.^12^ Interestingly, although there was no correlation between *OSM* expression and traditional clinical or laboratory markers of disease severity, they did find a correlation between intestinal *OSM* expression and need for early IBD surgery.^12^ In the investigation, patients that were grouped into an OSM^high^ expression module (based on the expression of OSM-associated genes) had a higher likelihood of primary nonresponse to anti-TNF therapy. They found only 10%–15% in the OSM^high^ module achieved complete (early) mucosal healing compared to 69%–85% of patients in the OSM^low^ module.^12^

Previous studies investigating baseline predictors of anti-TNF nonresponse have mainly focused on clinical (demographics, disease phenotype) characteristics and intestinal gene expression.^10, 11^ In a large cohort of adult-onset CD, Vermeire et al found older age, isolated ileitis, and previous history of CD surgery were associated with infliximab nonresponse.^3^ In addition, infliximab response has been associated with a c-reactive protein > 5 mg/L in adult-onset CD.^8^ In pediatric CD, favorable, early anti-TNF response has been associated with postinduction reductions in fecal calprotectin,^17^ improvements in wPCDAI,^24^ and higher infliximab concentrations^30–32^; however, none of these studies identified baseline laboratory tests associated with nonresponse. In addition, in a multivariable analysis of primary nonresponse conducted in a large cohort of 955 CD patients treated with infliximab, low infliximab concentration at week 14 (OR 0.35, 95% CI 0.20–0.62) was the only independent factor with a concentration of 7 µg/mL as the optimal week 14 drug target associated with remission at week 14 and week 54.^32^

More recently, smaller studies have evaluated blood biomarkers as predictors of CD complications (stricturing), however, not specific to anti-TNF response. In an observational study, Wu et al found elevated extracellular matrix protein 1, ASCA IgA, and CBir levels were associated future conversion from an inflammatory phenotype (B1) to a fibrostenotic phenotype (B2).^33^ Similarly, elevation of collagen type III alpha 1 chain and autoantibodies against colony-stimulating factor 2 were associated with development of intestinal strictures in pediatric patients with CD.^34^

To our knowledge, no other study has evaluated the relationship between pretreatment plasma OSM and outcomes with anti-TNF in IBD patients. Verstockt et al prospectively followed 54 IBD (24 CD and 30 UC) starting anti-TNF.^35^ They found anti-TNF endoscopic responders (in CD, SES-CD ≤ 2; in UC, Mayo endoscopic subscore ≤ 1) had a decrease in triggering receptor expressed on myeloid cells 1 (TREM1) expression from whole blood RNA with a similar reduction in mucosal *TREM1* and *OSM* gene expression (obtained from inflamed intestinal biopsies).^35^ Although the authors reported whole blood relative *OSM* expression (using qPCR) was higher (fold-change 0.67) in nonremitters, the relative *OSM* expression was not statistically different compared with remitters (*P* = 0.09). In the study, serum TREM1 was measured with ELISA, while OSM was quantified with whole blood gene expression. In our study, not only was the pretreatment plasma OSM associated with infliximab outcomes, but our results suggest that longitudinal OSM assessments may be beneficial for monitoring disease activity as persistent elevations in OSM beyond W12 were associated with continued CD inflammation at W52.

We have previously found that nCD64 strongly correlates with endoscopic-defined CD severity^14^ with *FCGRIA* expression >3-fold higher in the intestinal tissue of treatment-naive, newly diagnosed pediatric CD compared with controls.^13, 29^ Although nCD64 was shown to be effective in identifying silent CD (asymptomatic patients with ongoing intestinal inflammation who are at high risk of clinical relapse),^15^ we have not analyzed nCD64 as a companion diagnostic for anti-TNF therapy. However, one previous study found that colonic *FCGRIA* expression from the inflamed bowel was significantly upregulated in patients failing anti-TNF.^16^ To the best of our knowledge, this is the first study to show that elevations in pretreatment nCD64 (alone and in combination with elevated plasma OSM) were strongly associated with poor biochemical outcomes in patients receiving infliximab. Additional studies are ongoing to understand the potential mechanisms of increased nCD64 expression and anti-TNF nonresponse.

The strengths of the study include enrolling a prospectively monitored cohort of children and young adults with CD who predominantly received infliximab monotherapy in a real-world setting. Our goal was to evaluate pretreatment biomarkers with multiple treatment outcomes (including clinical and biochemical response) as it was not feasible to perform repeat endoscopy with our observational study. As plasma OSM has not been studied in the IBD population, we planned for multiple ROC curve analyses to identify potential OSM cut points for early/late biochemical nonresponse and nonremission. It is also important to highlight that our conclusions were based on plasma OSM obtained from a CD cohort that largely consisted of newly diagnosed CD patients as 65% of the patients started infliximab within 90 days of diagnosis.

Our primary outcome for this study was biochemical response utilizing fecal calprotectin as a surrogate for repeat endoscopy. Although there is little consensus and a paucity of data identifying fecal calprotectin cut points post-induction, we defined biochemical response as a >50% reduction in baseline fecal calprotectin as this cutoff was shown to reliably predict disease inactivity following infliximab induction in a pediatric cohort.^17^ Given the dynamic range of fecal calprotectin (50–2500 µg/g) for most commercial laboratories, it is vital to establish a reasonable cut point during induction for both fecal calprotectin and lactoferrin to prevent early anti-TNF discontinuation in patients who achieve clinical remission but continue to have mild elevations of the fecal biomarkers. Our study also highlighted the varying difficulty in obtaining a stool sample in CD patients despite the financial incentive (88% of the cohort returned fecal samples as scheduled) offered by the study.

## CONCLUSION

The Food and Drug Administration defines a companion diagnostic as a device that can identify patients most likely to benefit from a therapy or a device to monitor response with the purpose to adjust the treatment to achieve improved effectiveness. Although intestinal tissue companion diagnostics have previously been viewed as the preferred method to predict response,^10, 12, 36^ blood biomarkers are more convenient to collect in daily practice and much less invasive than colonoscopy. In conclusion, we showed pre-infliximab OSM^high^ is associated with early biochemical nonresponse and late (W52) clinical and biochemical nonremission. We also found that pretreatment nCD64^high^ was associated with biochemical nonremission. Although a validation study is warranted, it is reasonable for clinicians to evaluate pretreatment OSM and nCD64 prior to starting anti-TNF to determine those patients who may benefit from proactive therapeutic drug monitoring, higher starting doses, and more frequent disease activity monitoring.

## SUPPLEMENTARY DATA

Supplementary data are available at *Crohn’s & Colitis 360* online.

Supplemental Figure 1. We evaluated W12 infliximab concentrations between early (A) responders (>50% reduction in baseline fecal calprotectin) and nonresponders and (B) remitters (fecal calprotectin ≤ 250 µg/g) and nonremitters. (C) W12 infliximab concentration was also compared between pretreatment OSM^low^ and OSM^high^ patients. All comparisons were performed with the Mann-Whitney test.

otz026_suppl_Supplementary_Figure1

otz026_suppl_Supplemental_Figure_Legends

## Notes

Conference: We have presented the abstract from this study as an oral presentation at the 2019 Crohn’s and Colitis Congress in Las Vegas, NV, and as a poster presentation at the 2019 Digestive Diseases Week in San Diego, CA.

Supported by: This work was supported in part by the National Institute of Diabetes and Digestive and Kidney Diseases at the National Institutes of Health (NIH, K23 DK105229 to P.M., K23 DK094832 to M.J.R.), the Cincinnati Children’s Research Foundation Trustee Award Program (P.M.), the NIH P30 DK078392 (Gene Analysis Core) of the Digestive Disease Research Core Center in Cincinnati, and the Crohn’s and Colitis Foundation PRO-KIIDS Award (P.M.).

## References

[1] Hyams J, Crandall W, Kugathasan S, et al.; REACH Study Group Induction and maintenance infliximab therapy for the treatment of moderate-to-severe Crohn’s disease in children. Gastroenterology. 2007;132:863–873; quiz 1165. 17324398 10.1053/j.gastro.2006.12.003

[2] Hyams JS, Griffiths A, Markowitz J, et al. Safety and efficacy of adalimumab for moderate to severe Crohn’s disease in children. Gastroenterology. 2012;143:365–374.e2. 22562021 10.1053/j.gastro.2012.04.046

[3] Vermeire S, Louis E, Carbonez A, et al.; Belgian Group of Infliximab Expanded Access Program in Crohn’s Disease Demographic and clinical parameters influencing the short-term outcome of anti-tumor necrosis factor (infliximab) treatment in Crohn’s disease. Am J Gastroenterol. 2002;97:2357–2363. 12358256 10.1111/j.1572-0241.2002.05991.x

[4] Kelsen JR, Grossman AB, Pauly-Hubbard H, et al. Infliximab therapy in pediatric patients 7 years of age and younger. J Pediatr Gastroenterol Nutr. 2014;59:758–762. 25419596 10.1097/MPG.0000000000000533

[5] Colombel JF, Panaccione R, Bossuyt P, et al. Effect of tight control management on Crohn’s disease (CALM): a multicentre, randomised, controlled phase 3 trial. Lancet. 2018;390:2779–2789. 29096949 10.1016/S0140-6736(17)32641-7

[6] Papamichael K, Vajravelu RK, Vaughn BP, et al. Proactive infliximab monitoring following reactive testing is associated with better clinical outcomes than reactive testing alone in patients with inflammatory bowel disease. J Crohns Colitis. 2018;12:804–810. 29590345 10.1093/ecco-jcc/jjy039 PMC7189980

[7] Arnott ID, McNeill G, Satsangi J An analysis of factors influencing short-term and sustained response to infliximab treatment for Crohn’s disease. Aliment Pharmacol Ther. 2003;17:1451–1457. 12823146 10.1046/j.1365-2036.2003.01574.x

[8] Louis E, Vermeire S, Rutgeerts P, et al. A positive response to infliximab in Crohn disease: association with a higher systemic inflammation before treatment but not with -308 TNF gene polymorphism. Scand J Gastroenterol. 2002;37:818–824. 12190096

[9] Gaujoux R, Starosvetsky E, Maimon N, et al.; Israeli IBD research Network (IIRN) Cell-centred meta-analysis reveals baseline predictors of anti-TNFα non-response in biopsy and blood of patients with IBD. Gut. 2019;68:604–614. 29618496 10.1136/gutjnl-2017-315494 PMC6580771

[10] Arijs I, Quintens R, Van Lommel L, et al. Predictive value of epithelial gene expression profiles for response to infliximab in Crohn’s disease. Inflamm Bowel Dis. 2010;16:2090–2098. 20848504 10.1002/ibd.21301

[11] Kugathasan S, Denson LA, Walters TD, et al. Prediction of complicated disease course for children newly diagnosed with Crohn’s disease: a multicentre inception cohort study. Lancet. 2017;389:1710–1718. 28259484 10.1016/S0140-6736(17)30317-3 PMC5719489

[12] West NR, Hegazy AN, Owens BMJ, et al.; Oxford IBD Cohort Investigators Oncostatin M drives intestinal inflammation and predicts response to tumor necrosis factor-neutralizing therapy in patients with inflammatory bowel disease. Nat Med. 2017;23:579–589. 28368383 10.1038/nm.4307 PMC5420447

[13] Minar P, Haberman Y, Jurickova I, et al. Utility of neutrophil Fcγ receptor I (CD64) index as a biomarker for mucosal inflammation in pediatric Crohn’s disease. Inflamm Bowel Dis. 2014;20:1037–1048. 24788216 10.1097/MIB.0000000000000049 PMC4151275

[14] Minar P, Jackson K, Tsai YT, et al. Validation of neutrophil CD64 blood biomarkers to detect mucosal inflammation in pediatric Crohn’s disease. Inflamm Bowel Dis. 2017;24:198–208. 29272485 10.1093/ibd/izx022 PMC5831176

[15] Minar P, Jackson K, Tsai YT, et al. A low neutrophil CD64 index is associated with sustained remission during infliximab maintenance therapy. Inflamm Bowel Dis. 2016;22:2641–2647. 27749455 10.1097/MIB.0000000000000922 PMC5117809

[16] Wojtal KA, Rogler G, Scharl M, et al. Fc gamma receptor CD64 modulates the inhibitory activity of infliximab. PLoS One. 2012;7:e43361. 22937039 10.1371/journal.pone.0043361 PMC3427356

[17] Zubin G, Peter L Predicting endoscopic Crohn’s Disease activity before and after induction therapy in children: a comprehensive assessment of PCDAI, CRP, and fecal calprotectin. Inflamm Bowel Dis. 2015;21:1386–1391. 25851564 10.1097/MIB.0000000000000388 PMC4450968

[18] D’Haens G, Ferrante M, Vermeire S, et al. Fecal calprotectin is a surrogate marker for endoscopic lesions in inflammatory bowel disease. Inflamm Bowel Dis. 2012;18:2218–2224. 22344983 10.1002/ibd.22917

[19] Nancey S, Boschetti G, Moussata D, et al. Neopterin is a novel reliable fecal marker as accurate as calprotectin for predicting endoscopic disease activity in patients with inflammatory bowel diseases. Inflamm Bowel Dis. 2013;19:1043–1052. 23511035 10.1097/MIB.0b013e3182807577

[20] Turner D, Griffiths AM, Walters TD, et al. Mathematical weighting of the pediatric Crohn’s disease activity index (PCDAI) and comparison with its other short versions. Inflamm Bowel Dis. 2012;18:55–62. 21351206 10.1002/ibd.21649

[21] Turner D, Levine A, Walters TD, et al. Which PCDAI version best reflects intestinal inflammation in pediatric Crohn disease? J Pediatr Gastroenterol Nutr. 2017;64:254–260. 27050050 10.1097/MPG.0000000000001227

[22] Louis E Fecal calprotectin: towards a standardized use for inflammatory bowel disease management in routine practice. J Crohns Colitis. 2015;9:1–3. 25536671 10.1093/ecco-jcc/jju012

[23] Guiotto C, Daperno M, Frigerio F, et al. Clinical relevance and inter-test reliability of anti-infliximab antibodies and infliximab trough levels in patients with inflammatory bowel disease. Dig Liver Dis. 2016;48:138–143. 26614644 10.1016/j.dld.2015.10.023

[24] D’Arcangelo G, Oliva S, Dilillo A, et al. Predictors of long-term clinical and endoscopic remission in children with Crohn disease treated with infliximab. J Pediatr Gastroenterol Nutr. 2019;68:841–846. 30633110 10.1097/MPG.0000000000002262

[25] Kang B, Choi SY, Kim HS, et al. Mucosal healing in paediatric patients with moderate-to-severe luminal Crohn’s disease under combined immunosuppression: escalation versus early treatment. J Crohns Colitis. 2016;10:1279–1286. 27095752 10.1093/ecco-jcc/jjw086

[26] D’Haens G, Vermeire S, Lambrecht G, et al.; GETAID Increasing infliximab dose based on symptoms, biomarkers, and serum drug concentrations does not increase clinical, endoscopic, and corticosteroid-free remission in patients with active luminal Crohn’s disease. Gastroenterology. 2018;154:1343–1351.e1. 29317275 10.1053/j.gastro.2018.01.004

[27] Van Assche G, Vermeire S, Ballet V, et al. Switch to adalimumab in patients with Crohn’s disease controlled by maintenance infliximab: prospective randomised SWITCH trial. Gut. 2012;61:229–234. 21948942 10.1136/gutjnl-2011-300755

[28] Tanaka M, Miyajima A Oncostatin M, a multifunctional cytokine. Rev Physiol Biochem Pharmacol. 2003;149:39–52. 12811586 10.1007/s10254-003-0013-1

[29] Haberman Y, Tickle TL, Dexheimer PJ, et al. Pediatric Crohn disease patients exhibit specific ileal transcriptome and microbiome signature. J Clin Invest. 2014;124:3617–3633. 25003194 10.1172/JCI75436 PMC4109533

[30] Singh N, Rosenthal CJ, Melmed GY, et al. Early infliximab trough levels are associated with persistent remission in pediatric patients with inflammatory bowel disease. Inflamm Bowel Dis. 2014;20:1708–1713. 25153505 10.1097/MIB.0000000000000137

[31] Clarkston K, Tsai YT, Jackson K, et al. Development of infliximab target concentrations during induction in pediatric Crohn disease patients. J Pediatr Gastroenterol Nutr. 2019;69:68–74. 31232885 10.1097/MPG.0000000000002304 PMC6607916

[32] Kennedy NA, Heap GA, Green HD, et al.; UK Inflammatory Bowel Disease Pharmacogenetics Study Group Predictors of anti-TNF treatment failure in anti-TNF-naive patients with active luminal Crohn’s disease: a prospective, multicentre, cohort study. Lancet Gastroenterol Hepatol. 2019;4:341–353. 30824404 10.1016/S2468-1253(19)30012-3

[33] Wu J, Lubman DM, Kugathasan S, et al. Serum protein biomarkers of fibrosis aid in risk stratification of future stricturing complications in pediatric Crohn’s disease. Am J Gastroenterol. 2019;114:777–785. 31058681 10.14309/ajg.0000000000000237 PMC6532424

[34] Ballengee CR, Stidham RW, Liu C, et al. Association between plasma level of collagen type III alpha 1 chain and development of strictures in pediatric patients with Crohn’s disease. Clin Gastroenterol Hepatol. 2019;17: 1799–1806. 30213581 10.1016/j.cgh.2018.09.008 PMC6531351

[35] Verstockt B, Verstockt S, Dehairs J, et al. Low TREM1 expression in whole blood predicts anti-TNF response in inflammatory bowel disease. Ebiomedicine. 2019;40:733–742. 30685385 10.1016/j.ebiom.2019.01.027 PMC6413341

[36] Tew GW, Hackney JA, Gibbons D, et al. Association between response to etrolizumab and expression of integrin αE and granzyme A in colon biopsies of patients with ulcerative colitis. Gastroenterology. 2016;150:477–87.e9. 26522261 10.1053/j.gastro.2015.10.041

