# Supplementary figures and images for: Elevated Pretreatment Plasma Oncostatin M Is Associated With Poor Biochemical Response to Infliximab
Source: Crohns Colitis 360. 2019 Aug 19;1(3):otz026. doi: 10.1093/crocol/otz026 (PMC6798793; doi:10.1093/crocol/otz026)

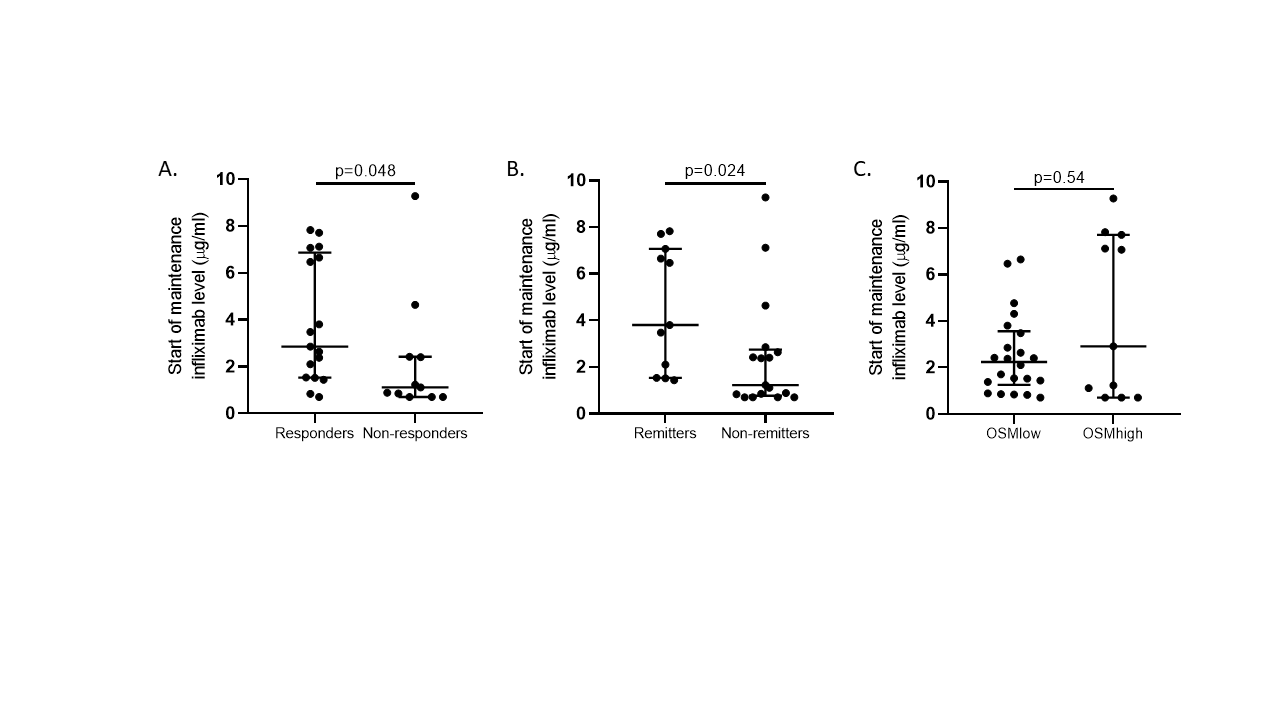

Supplement: otz026_suppl_Supplementary_Figure1 [file otz026_suppl_supplementary_figure1.png]
