# Supplementary material for: Elevated Pretreatment Plasma Oncostatin M Is Associated With Poor Biochemical Response to Infliximab
Source: Crohns Colitis 360. 2019 Aug 19;1(3):otz026. doi: 10.1093/crocol/otz026 (PMC6798793; doi:10.1093/crocol/otz026)
Supplement: otz026_suppl_Supplemental_Figure_Legends [file otz026_suppl_supplemental_figure_legends.docx]

**Supplemental Figure**

**Supplemental Figure 1**. We evaluated W12 infliximab concentrations between early (A) responders (>50% reduction in baseline fecal calprotectin) and non-responders and (B) remitters (fecal calprotectin ≤250 µg/g) and non-remitters. (C) W12 infliximab concentration was also compared between pre-treatment OSM^low^ and OSM^high^ patients. All comparisons were performed with the Mann-Whitney test.
